# Reference Jitter Values for Concentric Needle Electrode of Orbicularis Oculi and Frontalis Muscles Using Voluntary Activation Method in Sudanese Population

**DOI:** 10.1038/s41598-020-58037-z

**Published:** 2020-01-23

**Authors:** Afraa M. M. Musa, Ammar E. M. Ahmed

**Affiliations:** Department of Physiology, Faculty of Medicine, University of Khartoum, El Qasr Street, Khartoum, P.O. BOX 102 Sudan

**Keywords:** Neurophysiology, Neuromuscular disease

## Abstract

Single fibre electromyography is the most sensitive neurophysiological test for the diagnosis of neuromuscular junction disorders, particularly myasthenia gravis. The study aimed at establishing concentric needle (CN) normal jitter values for voluntarily activated orbicularis-oculi (V-OOc) & Frontalis (V-FRO) muscles in Sudanese population. 57 healthy volunteers (20 males & 37 females) were included in the study (mean Age 43.6 ± 14.2 years, range 18–70 years). V-OOc and V-FRO were tested in the same individual using CN. Jitter values were expressed as the mean consecutive difference (MCD) of 30 potential pairs in microseconds. The mean jitter, mean individual fibre pairs jitter & mean outliers jitter values with (upper 95% Confidence Limit-CL) for [OOc] were [26.9 ± 3.3 (31.97), 26.1 ± 8.9 (41.8) & 38.5 ± 5.7 (49.0) µs] & for [FRO] were [27.1 ± 3.0 (31.32), 26.4 ± 9.4 (42.9) & 39.9 ± 5 (49.2) µs] respectively. The suggested practical upper limits for mean jitter & for outliers were (32, 49 µs) for OOc & (31, 49 µs) for FRO. Our CN-jitter values were within the range of the few published studies. The study was unique in that it established and compared between CN reference jitter values of two voluntarily activated facial muscles (V-OOc & V-FRO) in the same individual in large number of healthy subjects.

## Introduction

Single-fibre Electromyography (SFEMG) is a special electromyographic test developed early in the nineteen sixties by the Swedish Scientists Stalberg and Ekstedt^1–3^. It is a valuable test used for the diagnosis of neuromuscular junction disorders, e.g. myasthenia gravis (MG)^4–6^. It is carried out using a specially designed single fibre needle (SF) electrode which can identify and record potentials from single individual muscle fibres. Expert neurophysiologists & equipped neurophysiology clinics are required to perform this safe but time-consuming test. Jitter is defined as the variability in the interpotential interval between two consecutive muscle fibre potentials of the same motor unit. Fluctuations in the time for endplate potentials to reach the action potential threshold at the neuromuscular junction produce most of this variation. Compared to Tensilon (edrophonium), repetitive nerve stimulation, and acetylcholine receptor antibody tests; jitter was proven to be the most sensitive test in the diagnosis of neuromuscular junction (NMJ) abnormalities^5,7–11^, yet not specific test as it was found to be increased in diseases of nerve (neuropathies) and muscle (myopathies)^6,12–18^.

Although SF electrode is the most accurate and highly selective in detecting potentials produced by individual muscle fibres, there are some disadvantages associated with the use of this needle electrode. These include the cost of the needle, electrodes sterilization, the time-consuming and technically difficult nature of the study and the risk of transmission of blood borne infectious agents, such as prion protein which is present in skeletal muscle^10,11^. Conventional sterilization methods cannot easily remove these proteins as it strongly adherent to metal surfaces, therefore reusable needle electrodes carry a risk of transmitting spongiform encephalopathies between humans^19^. Therefore, there is an increasing awareness about the use of the disposable, cheaper, less uncomfortable, readily available and easier to operate concentric needle (CN) and monopolar (MNP) electrodes worldwide^8,10,11,20–23^ instead of the standard SF electrode in jitter measurement for which normal jitter values had been established^24–27^.

With regard to ocular myasthenia gravis (OMG), jitter test (SFEMG) had been mostly carried out in either orbicularis oculi or frontalis muscles. Reviewing Literature, revealed one previous study carried out by Valls-Canals *et al*.^28^, who tested the two muscles together in the same individual using SF electrode on the electrically stimulated OOc and FRO muscles in 46 healthy subjects as a control for 20 patients with (OMG). They reported higher sensitivity of the stimulated SFEMG of the orbicularis oculi than the frontalis muscle in the diagnosis of OMG. Consequently, they recommended doing jitter first in the OOc muscle in patients with possible OMG as it may show negative results if only the frontalis muscle is examined. In contradistinction, the Japanese study carried out by Hiroko *et al*.^29^, who used the CN needle applying the voluntary activation method in 16 OMG patients, showed a slightly higher sensitivity of the FRO than the OOc.

## Justifications and Objectives

It is necessary to establish baseline reference SFEMG jitter values of the OOc & FRO because they are more easy to study, more sensitive in detecting abnormal jitter, and the earlier to be affected during the course of ocular myasthenia than limb muscles^18^. SFEMG was not only necessary for early diagnosis of patients with MG and other NMJ disorders, but also extremely important for carrying out an objective evaluation of their clinical progression and response to treatment^5^. In addition, it was extremely important to set normal baseline data for our Sudanese population taking into consideration that studies on jitter measurements had never been done in Sudan. Apart from two multicenter studies^18,30^ performed to establish concentric needle reference values for jitter measurements, a limited number of research articles addressed this issue, some of which targeted a small number of healthy subjects to serve as a control for myasthenic patients. Most of these studies tested only one facial muscle (either OOc or FRO). Axonal stimulation SFEMG method was performed testing the orbicularis oculi muscle^31,32^, while frontalis muscle was tested using the stimulation technique^33,34^. Regarding voluntary activation technique, it was performed on OOc^10,35,36^ as well as on FRO muscle^11,37^. Establishing upper limits for normal jitter values is essential for interpretation of patients’ test results.

The aim of this study was to establish and to compare normal (reference) concentric jitter values obtained performing voluntary activation technique in OOc & FRO muscles tested in the same control subject. Finally to find out the effect of age, gender, height and weight on CN-jitter values of voluntarily activated OOc & FRO in healthy subjects.

## Methods

The machine used to conduct SFEMG was Digital Medelec Synergy EMG (Electromyography) machine with a built-in SFEMG software used for recording and analysis. The analysis was done using extra Medelec Synergy Reader software. The recording was performed using CN with a red hub, diameter of 0.3 mm (30 G), recording area of 0.03 mm^2^ and needle length of 25 mm (TECA Accessories, Part No. X53153).

### Setting

This prospective cross-sectional analytic study was carried out in a period of one year, at the department of physiology, in the Faculty of Medicine of Khartoum University on healthy human volunteers who were selected randomly from the members and students of the Faculty in addition to healthy patients’ relatives accompanying patients referred to the Faculty neurophysiology clinic. The study recruited 57 normal subjects [37 of whom were females and 20 were males]. Their mean age (43.2 ± 14.0years) and their ages range (18–70 years). Only healthy Subjects were included in this study (i.e. they were subjected to thorough clinical examination). Subjects excluded from the study included those whose age was below 15 or above 70 years, diabetics, have thyroid or other autoimmune diseases, known to have a neuromuscular disease (neuropathy, NMJ disorders, myopathy), and on regular medication that is known to cause peripheral neuropathy or to affect NMJ transmission. Performing voluntary activation method, the OOc was investigated first using CN followed by the FRO muscle on the same subject at the same session. The OOc was tested in 55 out of 57 and the FRO in 52 out of 57 and both muscles were tested in 50 out of 57 control subjects.

### Muscle activation and recording methods

Jitter measurement was performed using CN instead of the standard SF electrode, the active electrode (G1) of which is an elliptical detection surface of the core located in the tip of the CN electrode. The outer diameter of the cannula represents the reference electrode (G2)^38^.

A high pass filter setting of 1–2 KHz for CN-jitter instead of the standard (500 Hz) used in SF recordings was recommended in many studies^8,10,11,22,35,36,39–41^. In this study, we used 1 KHz as a practical limit rather than 2 KHz as the signal amplitude would be higher resulting in an easier way to detect visually the summation of the single fibre action potentials (SFAPs) as recommended by Stalberg^18,35,40^. The low pass filter was set at 10 KHz, as for the SF^40^.

The amplitudes of the APs were optimized by slightly adjusting the electrode position in the best recording position for jitter measurements. Often, the spikes obtained with CN and MNP are not obtained from single muscle fibres so they are called apparent single fibre potentials, “aSFAP”^8^. Criteria recommended for CN recorded potentials to be accepted as SFAP is the same as for SF recordings except for the amplitude. Potential’s shape should be smooth, biphasic or triphasic (with initial positivity), stable and identical across a set of MU discharges with a sharp rising potential (rise time <300 µsec) and adequate amplitude (>100 µV) which is lower than the standard amplitude recommended for SF recordings (>200 µV)^10,11,18,35,36^.

Voluntary-activation method was performed by inserting CN into the muscle, usually in the middle third of its length, away from the end-plate zone (which lies somewhere near the midpoint of the muscle fibre) to record two or more time-locked muscle fibre potential (MFP) from the same motor unit during minimal voluntary contraction of the muscle^42^.

Concerning OOc, it was most easily studied laterally at its periphery (about 1 cm lateral to the canthus at various angles). OOc activation was controlled by asking the subject to close the eyes gently so as to avoid excessive blinking. Regarding FRO, it was best examined when the patient was lying. The needle was inserted to the forehead from a lateral position and activation was controlled by asking the subject to raise the eyebrows.

### Jitter calculation methods

Neuromuscular jitter represents the variation in the interpotential interval (IPI) between pairs of aSFAPs of the same motor unit in a voluntarily activated muscle.

Jitter was recorded from at least 20 different MFP pairs [the software in Medelec Synergy machine has the capability to record up to 30 MFP pairs] from different portions of the muscle, using up to three skin insertions at various angles. In addition, the software has the ability to create more than one pair from the single muscle fibre pair giving a total of more than 30 MFP pairs in one muscle. A constant detection position was maintained and the subject was asked to maintain a steady contraction while at least 50 discharges [up to 100] for each of the 20 single MFP pairs were collected. For jitter analysis, the examiners selected and accepted only individual potentials (spikes) with a minimal summation of many components (without notches or shoulders). The spike components used for analysis had a clear separation (150 µs) with no or minimal merging of one signal onto the other. Recordings were rejected by manual editing if overlapping potentials were present, less than 50 consecutive discharges were recorded or the interpotential interval was greater than 4 ms^38,42,43^.

Automatic measurements of the IPI were made between the green and yellow cursors adjusted manually by the examiner on a point relatively close to the baseline in the steep positive negative rising phase of the triggering potential and the corresponding point on succeeding or preceding jittering potential this is known as Amplitude level method of jitter measurement.

To minimize the influence of the slow variations in the IPIs, the jitter was expressed as the mean value of consecutive differences (MCD) of successive IPIs instead of using the standard deviation (SD). The MCD equation was then calculated automatically using the SFEMG software for each of the 30 different MFP pairs. Finally, the results of jitter measurements were presented for each muscle in individual subject as the average (mean) of the jitter values for the 30 pairs [all these calculation done automatically using the SFEMG software].

The effect of variable firing rates occur during voluntary activation was minimized by using the mean sorted-data difference-**(**MSD) instead of MCD. This again was done automatically using the SFEMG software, which calculates MCD and MSD values for each single MFP pair and chooses the smaller value as a measure of jitter for that pair.

All recordings were made and analyzed by the first examiner (Afraa.M.M.Musa) and revised by the second examiner (Prof. Ammar.E.M. Ahmed).

### Statistical analysis

Statistical analysis was performed using the Statistical Package for Social Science program (SPSS version 17). The mean jitter value for each individual muscle was calculated as the mean of individual jitter values of at least 20 a-SFAP pairs automatically using the machine. Then, the mean jitter of this muscle for the entire subject population was calculated [as the mean jitter of that muscle in all individuals** ± **SD]. The mean jitter + 2 SD [the 95% upper confidence limit] was considered as the upper practical limits of jitter for that muscle. In addition we calculated the mean individual fibre pair jitter value + 2 SD [the 95% upper confidence limit] as it was set by the (AD HOC committee of the AAEM single fibre special interest group) as the upper practical limits of normal for individual jitter values^24^. Finally, a new practical definition of abnormality includes the determination outlier values of individual fibre pairs which was introduced by Stalberg^35^. Ten percent of the recordings were allowed to be outside of the calculated limits. Therefore, the 90^th^ centile value was calculated for each set of 20 or more recordings. The 95% upper confidence limit (95% CL) of the 90^th^ centile value was calculated to define the outlier limit from which the upper practical limits for the individual fibre pair jitter values was estimated.

Correlation of jitter values with other parameters was done using the Pearson correlation test and comparison between the jitter values of OOc and FRO muscles in the same individual was done using paired student t-test.

### Ethical approval

All procedures performed in studies involving human participants were approved by the Ethics Committee of the Faculty of Medicine, University of Khartoum which was in accordance with the ethical standards of the national research committee and with the 1964 Helsinki declaration and its comparable ethical standards.

### Informed consent

Informed consent was obtained from all individual participants included in the study. Any participant had the right to leave the study at any time. Confidentiality and volunteer’s welfare were highly considered.

## Results

### Normal CN jitter values of voluntarily activated orbicularis oculi

**Figure 1:**
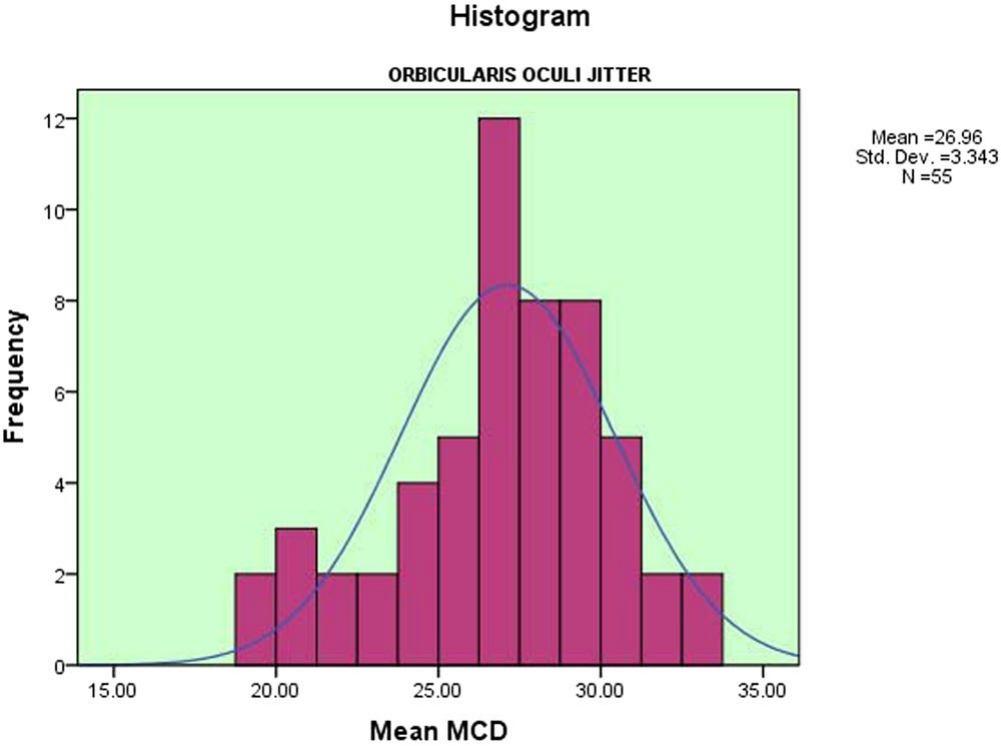
Distribution of Mean MCD Jitter Values of the Orbicularis Oculi Muscle. A histogram showing the distribution of CN- mean MCD jitter values of V-OOc muscle, in the form of mean, standard deviation and number of muscles tested.

**Figure 2:**
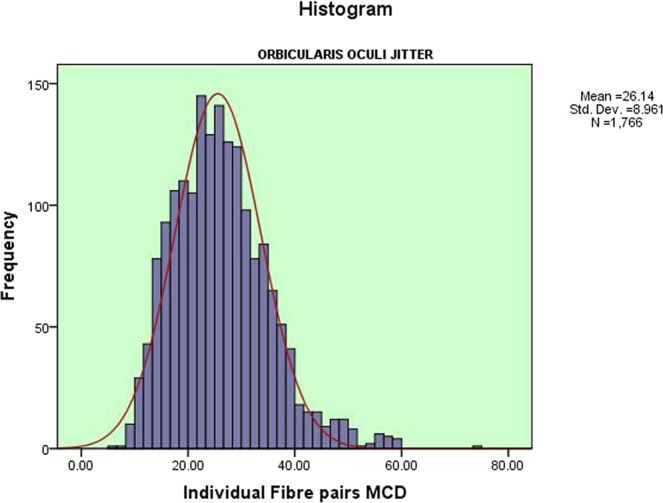
Distribution of Individual MCD Jitter Values of the Orbicularis Oculi Muscle. A histogram showing the distribution of CN- individual fibre pairs MCD jitter values of V-OOc muscle, in the form of mean, standard deviation and number of individual muscle fibre pairs tested.

The mean of the 55 V-OOc jitter values was 26.9 ± 3.3 µs (range 18.8–32.8 µs) as shown in [Fig. 1]. The upper 95% confidence limit was 32.0 µs. The mean of 1766 individual fibre pair jitter values was 26.1 ± 8.9 µs (range 6.6–59.9 µs) as shown in [Fig. 2]. The upper 95% confidence limit was 41.8 µs. The mean outlier values (the 90^th^ centile of individual fibre pair jitter values) for each subject was 38.5 ± 5.7 µs (range 24.6–50.0 µs), with an upper 95% confidence limit of 49.0 µs. There were no recordings with impulse blocking. The mean percent of recordings with abnormal fibre pairs ± SD was 1.7 ± 2.7 (range 0–10.9). The mean of MIPI values was 904.2 ± 258.9 µs (range 388.0–1653.1 µs).

### Normal CN jitter values of voluntarily activated frontalis

**Figure 3:**
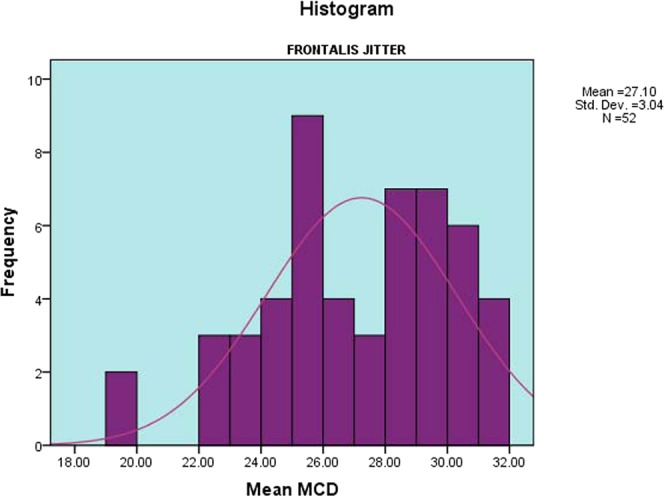
Distribution of Mean MCD Jitter Values of the Frontalis Muscle. A histogram showing the distribution of CN-jitter mean MCD jitter values of V-FRO muscle, in the form of the mean, standard deviation and number of muscles tested.

**Figure 4:**
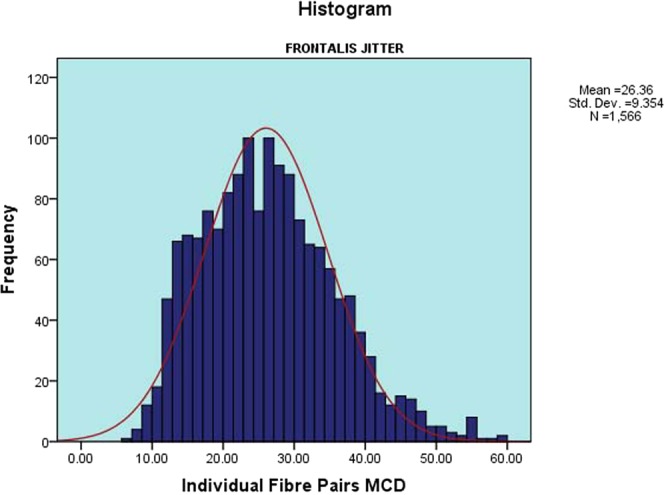
Distribution of Individual MCD Jitter Values of the Frontalis Muscle. A histogram showing the distribution of CN- individual fibre pairs MCD jitter values of V-FRO muscle, in the form of mean, standard deviation and number of individual muscle fibre pairs tested.

The mean of the 52 V-FRO jitter values was 27.1 ± 3.0 µs (range 19.3–31.7 µs) [Fig. 3]. The upper 95% confidence limit was 31.3 µs. The mean of 1566 individual jitter values was 26.4 ± 9.4 µs (range 7.1–59.0 µs) [Fig. 4]. The upper 95% confidence limit was 42.9 µs. The mean outlier values (the 90^th^ centile of individual fibre pair jitter values) for each subject was 39.9 ± 5.0 µs (range 29.8–54.5 µs), with an upper 95% confidence limit of 49.2 µs. There were no recordings with impulse blocking. The mean percent of recordings with abnormal fibre pairs ± SD was 1.2 ± 2.6 (range 0–11.1). The mean of MIPI values was 966.1 ± 419.6 µs (range 304.1–2524.4 µs).

### Demographic and anthropometric data and their Effect on jitter values of orbicularis oculi and frontalis

Fifty-seven control subjects were included in this study with a mean age of (43.6 ± 14.1 years), a weight of (66.7 ± 12.6 Kg) and a height of (164.9 ± 9.5 centimeters). The percentage of females comprised [65%] and males [35%] of the studied population.

There was no observed significant correlation (P > 0.05) between jitter values with age, weight, height or gender as shown in [Table 1].

**Table 1:** The Effect of Age, Weight, Height & Gender on Jitter Values of Orbicularis Oculi and Frontalis Muscles.

Muscle	Jitter/Age	Jitter/Weight	Jitter/Height	Jitter/Gender
	Paired correlation Sig. (2-tailed)			t-test/Sig
V-OOc	0.440	0.554	0.566	0.197
V-FRO	0.269	0.312	0.550	0.796

Significant P value ≤0.05.

### Comparison between CN-jitter values of voluntarily activated orbicularis oculi and frontalis in normal subjects

Comparison between the jitter values of V-OOc and V-FRO using CN revealed neither a significant statistical difference on paired t-test (P = 0.789) nor a significant correlation on a paired samples correlations (P = 0.169).

### Summary of our present results of jitter values compared with previous published data in the normal subjects

Summary of jitter values of this study compared to previous published data in normal subjects was illustrated in [Table 2].

**Table 2:** Summary of the Current Study Concentric Needle Jitter Values Compared to Previous Published Reference Values.

Needle	Muscle	No. of Subjects	Mean MCD per study (95% upper CL) in µs	No. of individual fibre pairs	Individual MCD values (95% upper CL) in µs	Outliers Jitter values (95% CL) in µs	Ref. No.
V-CN	OOc	55	26.9 ± 3.3 (32.0)≈32	1766	26.1 ± 8.9 (41.8)	38.5 ± 5.7 (49.0)≈49	**Current** **study**
V-CN	OOc	50	24.7 ± 3.1 (30.9)≈31	1000	24.7 ± 7.1 (38.9)	32.7 ± 4.1 (40.9)≈41	^35^
V-CN	OOc	10	26.5 ± 4.6 (35.7)[+2 SD]	238	26.1 ± 9.5 (49.7)[ + 2.5 SD]		^36^
V-CN	OOc	20	29.1 ± 3.9 (36.9)	484	28.8 ± 10.5 (49.8)		^10^
V-CN	OOc	92	22.9 ± 3.9 (31)	1,796		29.0 ± 7.6 (45)	^18^
V-CN	FRO	23		460	29.1 ± 12.0 (53.1)		^11^
V-CN	FRO	20	19.9 ± 2.9 (26.0)	400	19.9 ± 6.6	26.9 ± 4.4 (36.0)	^37^
V-CN	FRO	72	20.6 ± 3.6 (28.0)	1,622		25.8 ± 5.6 (38.0)	^18^
V-CN	FRO	52	27.1 ± 3.0 (31.3)≈31	1566	26.4 ± 9.4 (42.9)	39.9 ± 5.0 (49.2)≈49	**Current** **study**

### Summary of practical upper limit values for the mean jitter and jitter of individual fibre pair in orbicularis oculi and frontalis muscles

Our estimated values for upper 95% confidence limit (95% CL) of mean jitter, mean individual fibre pair jitter and mean outliers jitter [i.e. values above which the study should be considered abnormal] were summarized in [Table 3].

**Table 3:** Summary of Practical Upper Limit for the Mean MCD and for Individual MCD Jitter Values.

Needle	Method	Needle	Practical upper limit for mean MCD (µs)	Practical upper limit for individual MCD (µs)
*OOc*	Voluntary Activation	CN	31.97 ≈ **32**	49.0 ≈ **49**
*FRO*	Voluntary Activation	CN	31.32 ≈ **31**	**49.2** ≈ **49**

## Discussion

The present results of OOc and FRO CN-jitter values performed using the voluntary activation method in normal subjects, were compared with other CN-jitter studies^10,11,18,35–37^ and were summarized in [Table 2]. The limits for outliers are well defined for the current study and Stalberg studies^18,35,37^, more than the other studies. Most of the studies were performed in OOc, few tested FRO muscle.

Applying a similar methodology to^36^ and^10^, our OOc mean jitter values were well corresponding to the results of OOc in 10 healthy control subjects reported by Farrugia *et al*.^36^ and slightly lower than those obtained by Sarrigiannis^10^ who tested OOc in 20 normal control subjects. Similar to the current study, both studies^10,36^, used Medelec Synergy EMG machine and CN electrode size (0.3 mm diameter, a 0.03 mm2 recording area, Medelec Elite, X53153; Viasys Healthcare), the only difference was that they adopted high pass filtering of 2 KHz compared to 1 KHz in our study. The high pass filter was raised from 500 Hz to 2 KHz. Potentials were accepted if they fulfilled the criteria of SFAP and amplitude criterion of (100 μV) rather than the standard of (200 μV) because of increasing the high pass filtering reduced spike amplitudes. They used at least 20 MFP (at least 50–100 consecutive discharges for each pair), performing up to 3 needle insertions. Though the number of subjects was different between the three studies but using the same machine (SFEMG software), needle type and size, machine setting (filter, sensitivity,…etc.) and criteria for selecting the appropriate muscle fibre pair might explain results’ similarities. On the other hand, OOc jitter values of the current study were found to be slightly higher than those obtained by Kouyoumdjian and Stålberg^35^. They used a different machine (portable Keypoint Medtronic, Skovlunde, Denmark) with SFEMG software that uses Peak Method of jitter measurement compared to Medelec Synergy EMG which uses Amplitude Level method of jitter measurement. Another difference is that they used the smallest CN available (the Alpine DCF-25, Alpine BioMed, Skovlunde, Denmark, with 0.30 mm diameter and 0.019 mm^2^ recording area). Although both needles with a similar diameter (0.30 mm), their needle had smaller recording area (0.019 mm^2^) compared to the CN we used (0.03 mm^2^ recording area). They analyzed 20 different fibre pairs for calculation of jitter in each muscle compared to 30 or more in ours. The high pass filter setting was 1 KHz as we did. In spite of recruitment of a similar sample size (50 subjects compared to 55 in our study) and high pass filter setting (1 KHz); their use of different software, needle size and different numbers of individual MFPs analyzed might contribute to the slight variation observed between their results and ours.

Regarding CN jitter reference values for FRO muscle^11^, the results of V-FRO jitter values in this study were found to be somewhat lower than those established by Benatar *et al*.^11^ who aimed at estimating the accuracy of CN-SFEMG for the diagnosis of myasthenia gravis, putting into consideration that they used a similar Medelec Synergy machine and same concentric needle size. On the contrary, our FRO jitter values were higher than those obtained by Kouyoumdjian & Stalberg from 20 subjects using the voluntary contraction of the FRO muscle^37^.

Few researchers had applied different method of muscle activation (axonal stimulation technique) of SFEMG using CN^32,44^ or SF^28,31,32,44^ instead of voluntary activation method. They reported jitter values lower than those obtained using voluntary activation technique.

The cut-off values for individual MCD in the current study were calculated in the same way as recommended by Stalberg and colleagues in their multicenter study^18^. Our cut-off values [49 µs for both OOc and frontalis], were shown to be higher than those obtained by Stalberg and colleagues from 92 OOc studies {41 µs^35^ and 45 µs^18^} and 72 FRO studies {36 µs^37^, 38 µs^18^}. On the other hand, our values were lower than FRO values [56.8 μs] obtained in a Japanese multicenter^30^ which included a total of 69 normal subjects below the age of 60 years at six Japanese institutes. Their cut-off values for individual MCD were calculated using +2.5 SD or 95% CL of the upper 10th percentile MCD value^30^ which was different from the statics applied by Stalberg^18^.

Looking at the effect of age on OOc and FRO jitter values in Table 1, there was no significant correlation (p > 0.05) between jitter values and age similar to what was observed by other researchers on Extensor Digitorum and OOc muscles^22,23,35,39,41^. As well as this study revealed no significant effect of weight, height, and gender on jitter values of OOc and FRO muscles [Table 1].

Comparison between CN-jitter values of V-OOc and V-FRO showed no significant statistical difference and no significant correlation, as well as the cut-off values for mean MCD (32 vs. 31.3 µs) and individual MCD (49 vs. 49.2 µs) which were almost the same (Table 3). This was supported by Stalberg and colleagues who observed a small difference in the cut-off limits for V-OOc and V-FRO obtained during the multicenter study^18^.

There were similarities and dissimilarities between our findings and other researches. The cause of this variabilities in different studies might be attributed to the different techniques and equipment in different centers (including needle electrode type and size, filter setting, different software and machines which uses different algorithms for jitter measurement) in addition to different criteria for defining and selecting a fibre pair to be analyzed and included in the jitter measurement.

CN reference jitter values for voluntarily activated OOc and FRO, labeling the patient as having a positive jitter test, have been established in our Sudanese population. In fact, this study had provided practical upper limits of mean jitter and outliers CN-jitter values for V-OOc (32,49 μs) and (31,49 μs) for V-FRO. Age, gender, height, and weight did not show any effect on jitter values.

## Data Availability

The datasets analyzed during the current study are available from the corresponding author on reasonable request.
